# Epileptic high-frequency oscillations occur in neonates with a high risk for seizures

**DOI:** 10.3389/fneur.2022.1048629

**Published:** 2023-01-04

**Authors:** Nicola Kuhnke, Courtney J. Wusthoff, Eroshini Swarnalingam, Mina Yanoussi, Julia Jacobs

**Affiliations:** ^1^Department of Pediatric Neurology and Muscular Disease, University Medical Center, Freiburg, Germany; ^2^Stanford University, Palo Alto, CA, United States; ^3^Department of Pediatrics, University of Calgary, Alberta Children's Hospital, Calgary, AB, Canada

**Keywords:** high-frequency oscillations, Hypoxic Ischemia Encephalopathy (HIE), epilepsy, neonatal, biomarkers

## Abstract

**Introduction:**

Scalp high-frequency oscillations (HFOs, 80–250 Hz) are increasingly recognized as EEG markers of epileptic brain activity. It is, however, unclear what level of brain maturity is necessary to generate these oscillations. Many studies have reported the occurrence of scalp HFOs in children with a correlation between treatment success of epileptic seizures and the reduction of HFOs. More recent studies describe the reliable detection of HFOs on scalp EEG during the neonatal period.

**Methods:**

In the present study, continuous EEGs of 38 neonates at risk for seizures were analyzed visually for the scalp HFOs using 30 min of quiet sleep EEG. EEGs of 14 patients were of acceptable quality to analyze HFOs.

**Results:**

The average rate of HFOs was 0.34 ± 0.46/min. About 3.2% of HFOs occurred associated with epileptic spikes. HFOs were significantly more frequent in EEGs with abnormal vs. normal background activities (*p* = 0.005).

**Discussion:**

Neonatal brains are capable of generating HFOs. HFO could be a viable biomarker for neonates at risk of developing seizures. Our preliminary data suggest that HFOs mainly occur in those neonates who have altered background activity. Larger data sets are needed to conclude whether HFO occurrence is linked to seizure generation and whether this might predict the development of epilepsy.

## Introduction

High-frequency oscillations (HFOs) between 80 and 500 Hz are new markers identified in the human scalp and intracranial EEG. Physiological HFOs have been described in intracranial recordings and are linked to various cognitive processes, especially memory consolidation (1–3).

A large body of research has shown that the epileptic brain tissue also generates HFOs, which can be used as biomarkers for brain regions generating seizures (4). Many studies using intracranial EEG demonstrate that HFOs mainly occur inside the seizure onset zone, and several studies suggest that the removal of HFO-generating brain tissue correlates with post-surgical seizure outcome in patients with refractory epilepsy (5, 6). In addition, the amount of HFOs captured in an individual's EEG may reflect seizure propensity (7, 8). Early studies on epileptic HFOs suggested that these have very small generators preventing them from being visible on scalp EEG (9). Nevertheless, several investigators have identified epileptic HFOs on scalp EEG with a clear link to seizure onset areas (10, 11). Treatment response in different pediatric epilepsy syndromes has been linked to a reduction in HFOs (12). Simultaneous intracranial and scalp EEG recordings have proven a link between intracranial and scalp HFOs; simulation studies suggest that scalp HFOs are visible due to the low noise level in the high-frequency spectrum (13). The discovery of epileptic HFOs in scalp EEG substantially broadens the potential of these EEG markers and opens up an opportunity to investigate patients at risk of developing seizures. More recent studies even describe physiological HFOs in scalp recordings (14).

More than in any other age group, neonates are especially susceptible to seizures in the setting of acute brain injury. Among neonates with hypoxic-ischemic encephalopathy or stroke, 40–60% have acute seizures (15, 16) and 10–20% of these patients go on to develop chronic epilepsy with recurrent postneonatal seizures (17). It is unknown whether early treatment and suppression of symptomatic neonatal seizures can improve the overall outcome and reduce the likelihood of epileptogenesis. Multiple observational studies have shown that seizure burden is associated with a negative outcome even independent of other clinical variables, suggesting that the treatment of neonatal seizures may present an opportunity for intervention (15). A reliable biomarker for impending neonatal seizures would be highly valuable for evaluating interventions to prevent or modify these seizures.

The neonatal brain undergoes a rapid developmental change in the first week after birth. Maturation is reflected by changing EEG features during the neonatal period (18). It is now known that the neonatal brain is capable of producing HFOs, which can be detected on scalp EEG (19). The present study aimed to analyze scalp EEG from neonates at risk for seizures to determine whether HFOs are identifiable in this population.

## Methods

### Patients

All neonates who underwent continuous video EEG between November 2014 and September 2016 either at the Neuro-NICU of Lucile Packard Children's Hospital Stanford or at the NICU of the University Medical Center Freiburg were included. EEG recordings were initiated due to clinical concerns either for suspected seizure or a high-risk condition, such as hypoxic-ischemic encephalopathy. All EEG data were analyzed retrospectively. The study passed the local ethical review board at both University of Freiburg and Stanford. Included subjects had to have a long-term EEG performed due to clinical reasons, with a minimum of 30 min of EEG with low artifacts and a sampling rate ≥1,000 Hz. Medical records were used to extract clinical information, such as gestational age, birth weight, APGAR score, complications during pregnancy and birth, the severity of asphyxia or brain damage, hypothermia treatment, age at the time of EEG, and occurrence of seizures.

### EEG recording

Each recording was performed using 8–19 gold electrodes placed using adhesive paste. EEG was recorded continuously for at least 12 h in all patients. EEGs in Stanford were recorded using Nihon Kohden (Irvine, USA) with a 2,000 Hz sampling rate; and those in Freiburg with Micromed (Treviso, Italy) with a sampling rate of 2,048 Hz. A 30-min EEG segment was selected manually from a quiet sleep, with at least 2 h time separation from any seizures to sample the interictal period and a period selected to minimize artifacts (20, 21). EEGs were converted to EDF format using ASA software (ANT Neuro HQ, Enschede, the Netherlands).

EEGs were visually analyzed using a bipolar double banana montage. Clinical EEGs were scored according to the classification for Hypoxic Ischemic Encephalopathy (HIE) [Sarnat und (22)] (Normal; Moderately abnormal (Depression of amplitude <25 μV, periodic or paroxysmal); Severely abnormal (Periodic or isoelectric). A similar classification could not be made with the other etiologies, as a standardized conceptualization of EEG severity is not currently available for pathologies other than HIE. EEGs with obscuring focal or diffuse artifacts were excluded.

### HFO analysis

**Figure 1:**
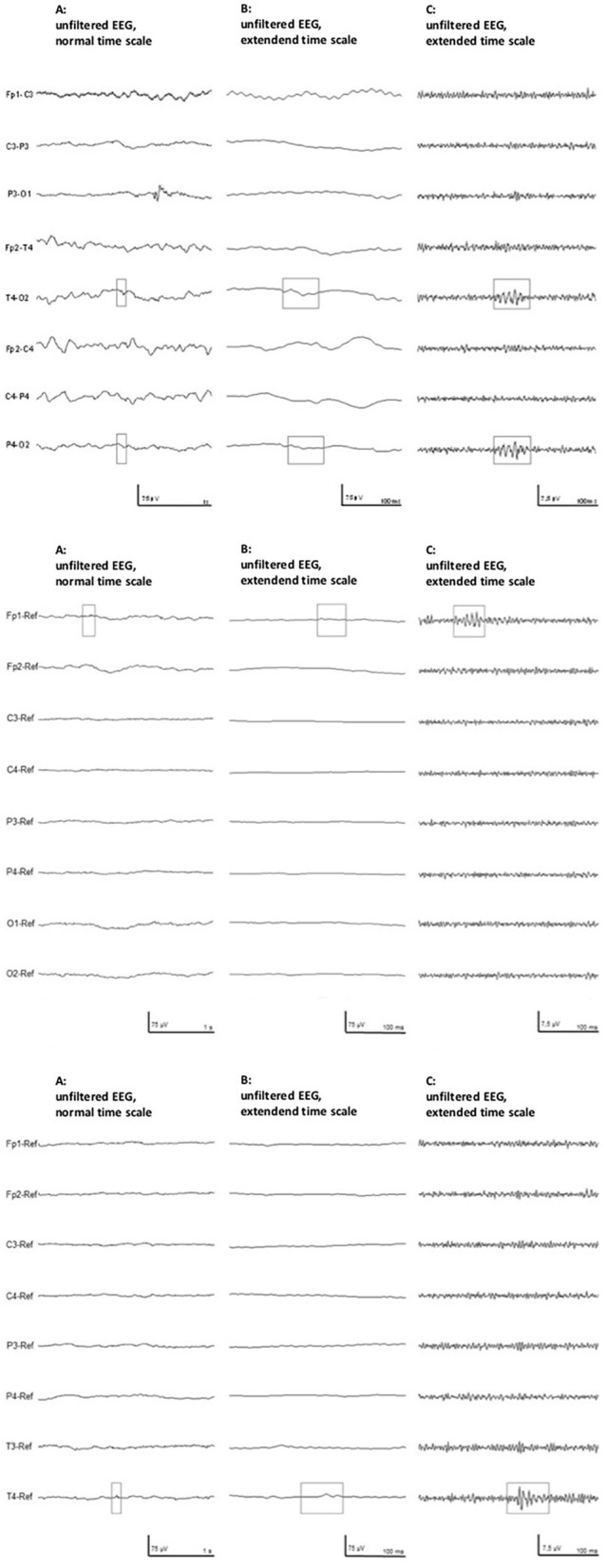
Examples of three identified HFOs in neonates. **(Left)** Unfiltered EEG with a normal time scale, **(Middle)** unfiltered EEG with an extended time scale, and **(Right)** the high-pass filtered EEG with an extended time scale. The top row shows an example from patient #1, the middle row from patient #9, and bottom row shows data from patient #11.

**Figure 2:**
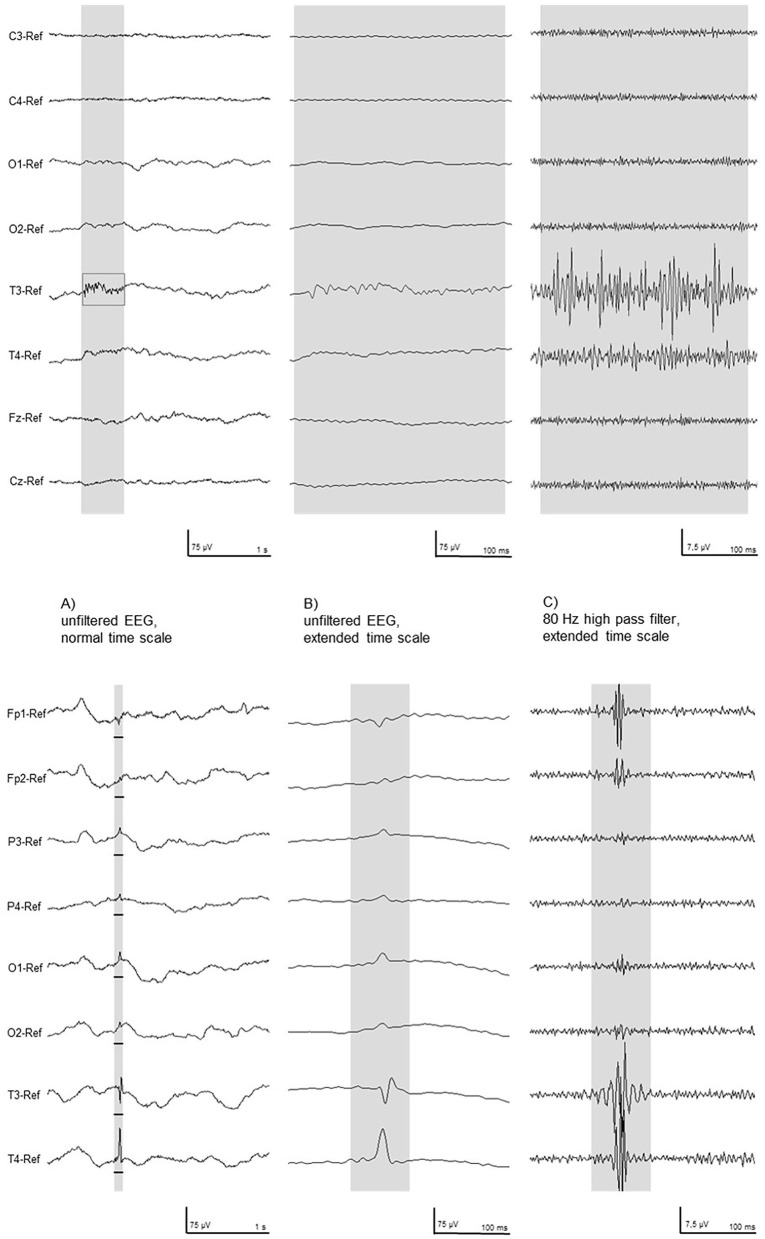
Two typical examples of high-frequency activity in the filtered EEG **(Right)** were excluded as artifact, as seen in the unfiltered EEG **(Left)**. These EEG segments were recorded in patient # 10.

HFOs were visually identified using Stellate Harmonie Systems (Stellate, Montreal, Canada), as previously described (23). EEG was displayed with a split screen, visualizing the high-pass filtered EEG for HFO analysis, and an EEG with standard filter setup (HFF: 70 Hz, LFF: 1 Hz, and Notch filter: 60 Hz) for spike and artifact identification. For HFO identification, a FIR high-pass filter of 80 Hz was applied and the maximally possible time resolution of 0.6 s/page was selected. All HFO markings were performed visually by two independent reviewers. Only events which were identified by both were taken into the analysis. HFOs were identified as such if the oscillation had a significantly higher amplitude than the background EEG and consisted of at least four oscillations (see Figure 1). In the case of an associated artifact in the unfiltered EEG on the same or a neighboring channel, HFOs occurring simultaneously were excluded (see Figure 2). Rates of spikes and HFOs were calculated for each channel. The rate of HFO occurrence was compared between the etiological cohorts of asphyxia vs. non-asphyxia related etiologies.

### Statistical analysis

Statistical comparisons of HFO rates between patients with different EEG background patterns as well as between different etiologies were performed through a Mann–Whitney *U*-test. The significance level was defined at *p* < 0.05. All statistics used SPSS software.

## Results

Thirty-eight neonates had continuous EEG meeting the inclusion criteria during the study period, but in 24 subjects, high-frequency artifacts were present during the recording. One major cause of these artifacts was electrical interference from the simultaneous use of a stand-alone aEEG machine. Fourteen patients had EEG that could be used for detailed analysis. The average gestational age of our patient population was 38 + 1 weeks with an average birth weight of 3,035.2 g. Of that, 57% were male. The average age of the patients at the time of obtaining the EEG was 9.6 days. Clinical and electrographic seizures were present in 11/14 (78.5%) patients. Asphyxia was the reason to obtain an EEG in 4 out of 14 patients with one patient diagnosed with both perinatal stroke and asphyxia. Neonatal seizures were the reason for EEG acquisition in 4 out of 14 and a vascular event occurred in 3 out of 14. Two patients had a confirmed underlying genetic etiology. The clinical characteristics of these patients are summarized in Table 1.

**Table 1:** Clinical details of all analyzed patients.

**Patient #**	**M/F**	**Week of gestation**	**Birthweight**	**Age at EEG**	**APGAR**	**Reason for EEG/risk**	**Seizure**	**Mode of delivery**
1	M	38 + 2	2,952 g	2 d	5′8/10′9	Neonatal seizures	Yes	CS
2	F	40 + 0	3,200 g	12 d	5′9/10′9	Neonatal seizures	Yes	VD
3	F	39 + 1	3,400 g	4 d	5′9/10′9	Intracranial hemorrhage	Yes	VD
4	M	39 + 2	3,598 g	20 d	5′5/10′8	Genetic epilepsy/KCNQ2	Yes	CS
5	F	40 + 2	3,349 g	22 d	5′8/10′9	Genetic epilepsy/STXBP1	Yes	VD
6	M	40 + 4	2,740 g	3 d	5′7/10′8	Neonatal seizures	Yes	CS
7	F	40 + 0	3,245 g	1 d	5′4/10′8	Perinatal media infarction	Yes	CS
8	M	40 + 5	3,520 g	1 d	5′8/10′9	Bilateral stroke	Yes	CS
9	M	39 + 0	3,770 g	4 d	5′8/10′9	Neonatal seizures	Yes	CS
10	M	39 + 4	3,620 g	1 d	5′5/10′7	Asphyxia	No	VD
11	M	24 + 0	700 g	2 m	5′4/10′7	Asphyxia	No	CS
12	F	37 + 0	2,200 g	2 d	5′9/10′9	Asphyxia	Yes	CS
13	M	38 + 6	3,400 g	2 d	5′8/10′9	Asphyxia/stroke	Yes	VD
14	F	37 + 6	2,800 g	1 d	5′3/10′5	Ashyxia	No	CS

The term neonatal seizures was used if clinically present seizures were seen without known underlying cause and with normal MRI.

CS, cesarean section; f, female; d, days; m, months; M, male; VD, vaginal delivery.

In seven patients, the EEG background activity was classified as normal; in six patients, moderately abnormal; and in one patient, severely abnormal.

**Figure 3:**
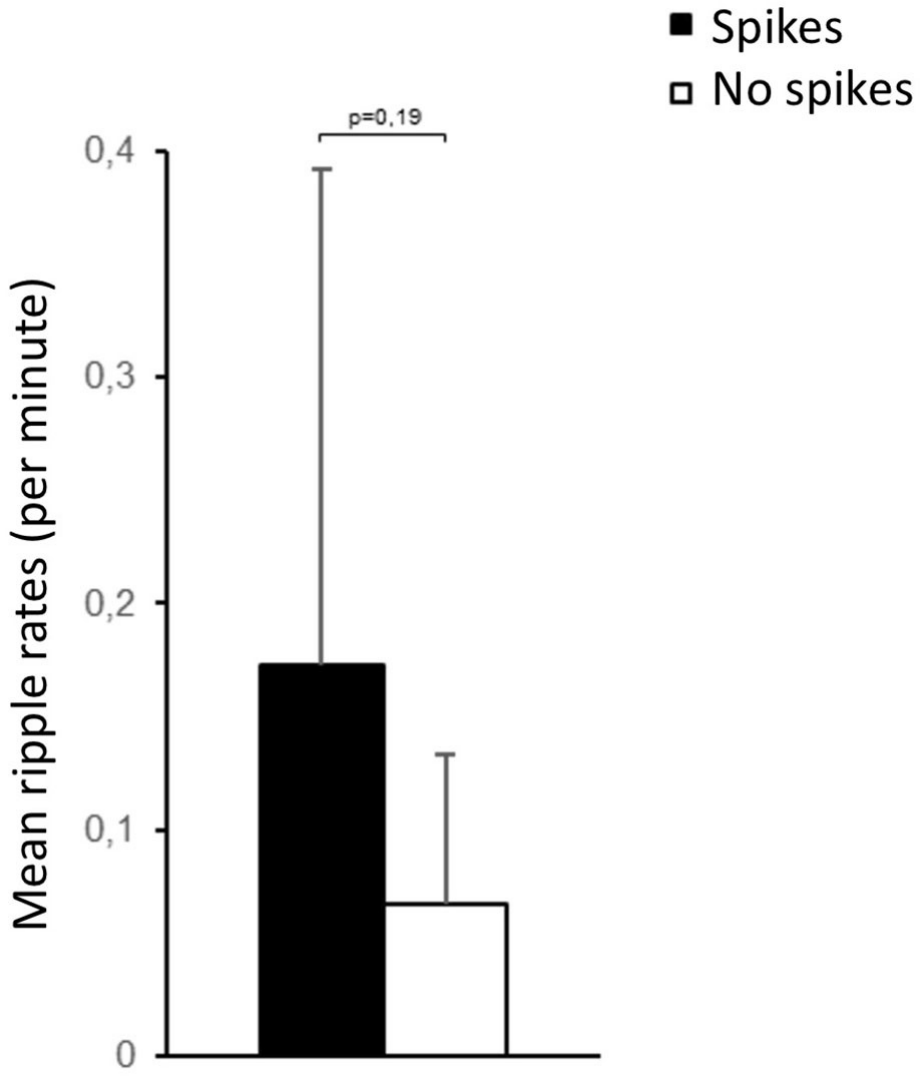
No significant difference in HFO rates was seen for channels with and without interictal epileptic spikes.

A total of 241 EEG channels could be analyzed. Epileptic spikes were seen in 11 patients. The average rate of spikes was 0.72 ± 0.96/min. HFOs could also be identified in 11 patients (Figure 1). The average rate of HFOs was 0.34 ± 0.46/min. Approximately 3.2% of HFOs occurred at the same time as epileptic spikes. There was no significant difference in HFO rates among patients that showed spikes and those who did not (Figure 3). The three neonates without HFO did not significantly differ from their counterparts either clinically or neurophysiologically.

HFO and spike rates for each patient are presented in Table 2.

**Table 2:** Details of EEG results. Rates are given as the length of analyzed EEG was variable due to artifacts.

**Patient #**	**EEG background**	**# of spikes**	**Spike rate/min**	**# of Ripples**	**Ripple rate/min**
1	Normal	80	2.67	4	0.13
2	Normal	8	0.27	8	0.27
3	Moderatly abnormal	8	0.28	47	1.57
4	Normal	30	1	6	0.2
5	Normal	0	0	4	0.13
6	Moderatly abnorm	16	0.53	33	1.1
7	Normal	76	2.53	8	0.27
8	Normal	3	0.1	0	0
9	Moderatly abnormal	17	0.57	17	0.57
10	Moderatly abnormal	0	0	8	0.23
11	Normal	4	0.13	2	0.07
12	Moderatly abnormal	61	2.03	0	0
13	Moderatly abnorm	7	0.23	4	0.13
14	Severly abnormal	0	0	0	0

**Figure 4:**
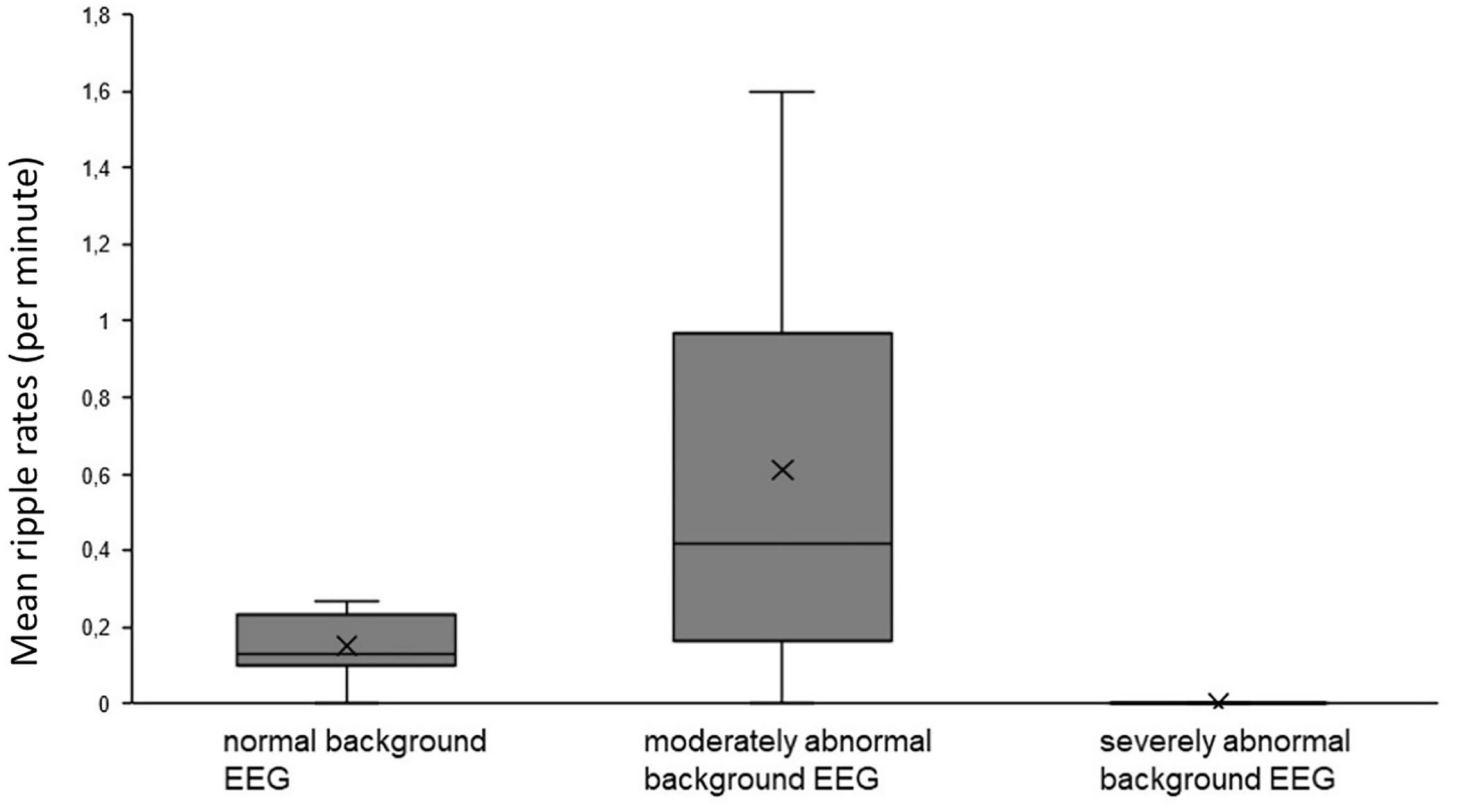
No significant difference in HFO rates was seen in patients with abnormal background activity compared to those with normal background activity.

For epileptic spikes, no significant differences were found for different background activity, seizure activity, or etiology. HFO rates showed a tendency toward higher HFO rates (0.61 ± 0.57/min) in patients with a moderately abnormal background EEG in comparison to normal background EEG (0.15 ± 0.09/min; Mann–Whitney *U*-test *p* = 0.169, Figure 4). However, there was a significant difference in HFO rates between patients with normal vs. abnormal EEG backgrounds (*p* = 0.005). There were no significant differences in HFO rates depending on the occurrence of seizures. No differences were found in HFO rates comparing the group of patients with abnormal MRI and the group of neonatal seizures without MRI abnormality.

## Discussion

In this study, HFOs were clearly identified on scalp EEG recorded in the neonatal period. All patients under investigation belonged to a high-risk population, and therefore, no conclusion can be drawn in the presence of physiological HFOs in the healthy neonatal brain. The association between abnormal background and higher rates of HFOs suggests that the identified HFOs might be a sign of abnormal function.

Recording EEGs in an ICU environment is often challenging due to the multiple other electronic devices necessary to support and monitor the neonate. In this retrospective data analysis, we had to exclude more than half of the recorded EEGs due to continuous artifacts in the EEG recordings. Some of these artifacts such as ventilation and ECG artifact are obvious and have been described previously (24). In the analysis of high-frequency components in the EEG, more subtle interferences might become visible, which usually do not prevent the clinical analysis of the EEG data. In our case, most prominently continuous high-frequency noise was a result of a separate aEEG machine being concurrently used during EEG recording. Unfortunately, due to the retrospective design of this study, this problem could not be addressed, resulting in a relatively low number of neonates that could be included. Future studies might benefit from the use of combined EEG–aEEG machines as well as low-noise amplifiers that have been proven to increase the visibility of HFO in intracranial EEG (25).

HFO analysis in scalp EEG cannot be performed automatically, as designed detectors are vulnerable to high noise artifacts and have low specificity (13). For this reason, all HFO markings in this study were performed visually by two reviewers. Only events, which were identified by both, were taken into the analysis. At all times, events that co-occurred with artifacts in the unfiltered EEG were excluded, as suggested by Andrade-Valenca and coauthors (10). This might have led to the exclusion of some real HFO events and explain the lower rate of HFO found in the present study compared with other pediatrics studies (26, 27). Even if the EEG sampling rate would have allowed us to look at frequencies above 250 Hz, we limited our analysis to the ripple frequency range as this has been most often described on scalp EEG (4, 28).

Using a higher density of electrodes has shown to be more sensitive to detecting HFOs (23, 29). However, in the neonatal population, this may come with certain disadvantages, such as the risk of breakage of the fragile neonatal skin. We utilized eight electrodes in two of our patients, while the rest had a set of 18 electrodes for EEG acquisition. However, there was no significant difference in HFO detection between these two groups. Therefore, following the standard of practice, which is to utilize a reduced neonatal montage in scalp EEG acquisition, will likely have no major impact on HFO detection.

In our study, we included all consecutive patients within a 2-year period who underwent continuous EEG monitoring in a NICU setting. The clinical spectrum of disease varied largely, including patients with genetic and structural reasons for developing seizures. No difference in the rate of HFO occurrence was found in different etiological subgroups, mainly between the asphyxia and non-asphyxia groups, which, if replicated in larger studies, may suggest that HFOs are a ubiquitous marker for pathological or epileptic brain activity rather than specific for certain pathology. This is in line with intracranial EEG data recorded from different types of lesions as well as scalp EEG data recorded in epileptic encephalopathies with varying underlying diseases (30–33). A much larger cohort of neonates should be studied to understand under which circumstances HFOs occur in the neonatal brain.

It is also worth mentioning that various central nervous system medications including anti-seizure medications as well as therapeutic hypothermia itself can alter the HFO rates (34). In our patient population, EEG was initiated in all, upon clinical suspicion and before starting anti-seizure medication. However, the potential effect of concomitant sedative medication and therapeutic hypothermia on HFO rates cannot be excluded. It would be useful to further evaluate the impact of concurrent therapies on HFO rates in larger cohorts with etiological heterogeneity.

The main finding of the present study is that the neonatal brain is capable of generating discrete HFOs. The mechanisms of HFO generation are still not completely understood; it has been hypothesized that epileptic HFOs mainly reflect principal cell action potentials in which pathological synchronization of fast-firing interconnected neurons leads to the formation of high-frequency population spikes (35). This mechanism requires a relatively complex neuronal network, which seems to be already in place within the first 4 weeks of life. Simultaneous intracranial and scalp EEG are the strongest proof of the visibility of HFOs on scalp EEG but are ofcourse limited to patients with refractory epilepsy (11, 21). The high skull conductivity of children might increase the visibility of scalp HFOs in the younger population (26). Some prior studies have suggested that HFOs most often are seen in scalp EEG and also show many interictal epileptic spikes (36). In the current study, epileptic spikes were less frequent than have been reported in older pediatric children with seizures (37, 38). In the current study, the observed HFOs were not co-occurring with epileptic spikes as has been described in other age groups. Data in adults suggest that up to 50% of scalp HFOs occur during spikes; the generation of both is not necessarily linked (36). Detailed analyses suggest that HFOs often precede spikes, though not consistently, supporting a hypothesis that there are independent underlying mechanisms for these phenomena (39). This seems to be even more the case in neonates, for whom spikes are not epileptiform (40, 41).

Neonatal brain injury is often associated with acute seizures; seizure burden is associated with long-term outcomes (42, 43). Up to 20% of affected neonates develop chronic epilepsy (15). A reliable non-invasive biomarker for epileptic activity in the neonatal brain would be useful. A previous study has demonstrated that the presence of HFOs can predict the development of epilepsy in older children presenting with their first seizure (44). This study is not able to make a similar inference; however, it shows that HFOs can be identified in neonates and may be associated with pathologic brain activity. Future studies are needed to investigate larger groups of healthy neonates as well as those with different underlying pathologies to further clarify the significance of HFOs in these patients.

## Data availability statement

The original contributions presented in the study are included in the article/supplementary material, further inquiries can be directed to the corresponding author.

## Author contributions

All authors listed have made a substantial, direct, and intellectual contribution to the work and approved it for publication.
